# Growth response of greenhouse-produced muskmelon and tomato to sub-surface drip irrigation and soil aeration management factors

**DOI:** 10.1186/s12870-020-02346-y

**Published:** 2020-04-06

**Authors:** Yuan Li, Wenquan Niu, Xiaoshu Cao, Mingzhi Zhang, Jingwei Wang, Zhenxing Zhang

**Affiliations:** 1 Northwest Land and Resources Research Center, Shaanxi Normal University, Xi’an, 710119 Shaanxi China; 2 Institute of Soil and Water Conservation, Northwest A&F University, Yangling, 712100 Shaanxi China; 3 Institute of Water-saving Agriculture in Arid Areas of China (IWSA), Northwest A&F University, Yangling, 712100 Shaanxi China; 4 Institute of Soil and Water Conservation, Chinese Academy of Sciences & Ministry of Water Resources, No.26 Xinong Road, Yangling, 712100 Shaanxi China; 5 College of Water Resources and Hydropower, State Key Laboratory Base of Eco-Hydraulic Engineering in Arid Area, Xi ‘an University of Technology, Xi’an, 710048 China; 6 Henan Provincial Water Conservancy Research Institute, Zhengzhou, 450000 China; 7 College of Resources and Environment, Shanxi University of Finance and Economics, Taiyuan, 030006 Shanxi China; 8 Key Laboratory of Vegetation Ecology, Ministry of Education, Institute of Grassland Science, Northeast Normal University, Changchun, 130024 Jilin Province China; 9 State Environmental Protection Key Laboratory of Wetland Ecology and Vegetation Restoration, School of Environment, Northeast Normal University, Changchun, 130117 Jilin Province China

**Keywords:** Supplemental soil aeration, Leaf area index, Dry matter partitioning, Muskmelon, Tomato

## Abstract

**Background:**

Hypoxia causes injury and yield loss. Soil aeration has been reported to accelerate the growth of plants and increase crop yield. The aim of this study was to examine growth response of greenhouse-produced muskmelon to 3 levels of sub-surface drip irrigation (I), 3 different installation depths of drip laterals in the soil (D), and 4 levels of supplemental soil aeration frequency (A). A fractional factorial experiment was designed to examine these treatment effects on marketable fresh fruit yield, leaf area index during 3 growth stages, and dry matter partitioning at harvest. In addition, we studied the response of fruit yield and dry matter of tomato to 2 levels of burial depths of subsurface tubing in combination with 3 frequency levels of soil aeration.

**Results:**

Results showed that soil aeration can positively influence the yield, leaf area index, dry matter and irrigation use efficiency of the muskmelon (*p* < 0.05). The fruit yield of muskmelon and tomato were increased by 21.5 and 30.8% respectively with 1-d and 2-d aeration intervals compared with the no aeration treatment.

**Conclusions:**

The results suggest that soil aeration can positively impact the plant root zone environment and more benefits can be obtained with aeration for both muskmelon and tomato plants.

## Background

Use of sub-surface drip irrigation under protective structures to produce high-value vegetables for the fresh food market is increasing in the semi-arid areas of Northwest China. These production technologies significantly improve water use efficiency compared to traditional spray or furrow irrigation systems [1–3]. The topsoil water content depends on the sub-surface drip application frequency, rate, depths, leakage and evapotranspiration. Manufacturer can vary the sub-surface depth of the buried drip tubing to facilitate cultivation along with the rate, amount, and frequency of irrigation to control the wetted portion of the root zone and optimize soil water availability and accessibility to the plant root system [4–6].

In addition to soil water, rhizosphere O_2_ content is one of the most important factor affecting respire, grow, develop, and function normally of plant roots [5, 7]. Most of O_2_ supply is obtained directly by continuous diffusive air exchange between the atmosphere and soil [8]. Previous studies have shown that hypoxia stress limited leaf growth, the rate of leaf expansion declined, and the size of leaf reduced [9]. High CO_2_ concentrations (at 2500 μL L^− 1^) or low (10% by volume) O_2_ in the root-zone significantly increased the activity of lactate dehydrogenase (LDH), alcohol dehydrogenase (ADH), and pyruvate decarboxylase (PDC) in muskmelon plants, resulting in inhibition of growth, and decrease in fruit yield and quality [10–12].

Subsurface drip irrigation allows the application of air and soluble materials (such as O_2_, nutrients, pesticide) directly to the root zone. Studies have shown that these practices can be very useful in overcoming problems associated with hypoxia in the root zone of irrigated crops over a range of crops, soil water contents and soil types, and for improving crop performance under oxygen-deficient conditions [13–15]. Aerated irrigation positively affected yield and improved quality for cotton, beans, squash, pumpkin, cucumber, tomato, rice and other crops especially on heavy and saline soils [16–20].

These demonstrated positive effects of aerated irrigation and supplemental aeration on growth and yield have not been completely explained in the literature. It is quite possible that improved LAI of the leaves may be a key mechanism for the increased yield and quality. For given ambient conditions (light, temperature, water, nutrition) crop growth and yield is directly related to LAI. Higher LAI, photosynthetic rate and chlorophyll content are key indicators and predictors of higher crop yields [21]. However, it is not known how changing sub-surface irrigation practices (tubing placement depth, irrigation level and frequency) and artificial aeration frequency would affect LAI.

We suspect that varying the aeration frequency, aeration volume, aeration position and irrigation amount would result in a possible change in rhizosphere soil environment, LAI, and irrigation use efficiency of plants, thus changing the plant growth and fruit output. The specific objective of this study was to determine the response of greenhouse-produced muskmelon and tomato plants on clay loam soils of the semi-arid area of Northwest China under various combinations of sub-surface irrigation volumes, tubing placement depth, and supplemental aeration frequency. Fresh fruit yield, leaf area index, and dry matter partitioning were taken as measures of response to these treatment factors.

## Results

### Muskmelon results

#### LAI of muskmelon

Values in the LAI_25_ row of Table 1 corresponded to the tendril elongation growth period. The 55 DAT (LAI_55_) and 75 DAT (LAI_75_) measurements were taken during the flowering and at fruit harvest. The overall mean of 2.69 at 40 DAT was close to double the value of 1.31 at 25 DAT. At 75 DAT the LAI of 2.86 was only about 4% higher than at 40 DAT. The 10 cm tubing placement depth tended to be significantly lower compared to 25 and 40 cm depth for the 55 and 75 DAT measurements. Differences between the LAI marginal means for irrigation level were non-significant for all 3 sampling dates (Table 1). Among the single-factor analyses, tubing placement depth had a significant impact on both LAI_55_ and LAI_75_, aeration frequency had an extremely significant impact on LAI_25_. The identified significant D x A interaction for LAI_25_, and A x I interaction for LAI during the tendril elongation growth period and fruit harvest period.

**Table 1:** Marginal means and 2-factor interactions for marketable fruit yield, LAI, post-harvest dry matter partitioning, and irrigation use efficiency response of greenhouse-produced muskmelon plants to sub-surface drip-irrigation tubing placement depth x soil aeration frequency x irrigation level treatment factors. In each row, the means for the levels of a given factor not followed by the same letter are significantly different at the 5% level. Two-factor interaction is significant at the 1% (**), 5% (*), or not significant (ns) level

Response	Tubing Placement Depth (D _cm_)			Soil Aeration Frequency (A _interval in day_)				Irrigation Level (I _to % field capacity_)			F-value					
	D_10_	D_25_	D_40_	A_1_	A_2_	A_4_	A_N_	I_70_	I_80_	I_90_	D	A	I	DxA	DxI	AxI
Yield (kg)	1.48ab	1.59a	1.38b	1.64a	1.51ab	1.43b	1.35b	1.53a	1.51a	1.40a	3.012 ns	1.995 ns	0.753 ns	0.296 ns	3.606*	0.988 ns
LAI_25_	1.41a	1.41a	1.11a	1.63a	1.42a	1.33a	0.84b	1.38a	1.41a	1.13a	1.128 ns	8.087**	1.608 ns	3.018*	1.143 ns	2.674 ns
LAI_55_	2.22b	3.05a	2.81a	3.11a	2.55a	2.62a	2.48a	2.52a	2.61a	2.94a	5.334**	1.028 ns	2.029 ns	1.486 ns	1.792 ns	1.623 ns
LAI_75_	2.31b	3.25a	3.02a	3.00a	3.06a	2.96a	2.43a	2.63a	3.02a	2.94a	5.788**	1.201 ns	0.777 ns	1.017 ns	0.986 ns	2.703*
Total DM (g)	43.3b	52.9ab	60.3a	62.6a	54.2ab	49.2b	42.6b	52.2a	51.6a	52.6a	5.347**	3.900*	0.017 ns	0.440 ns	3.821*	2.659*
Stem DM (g)	11.3b	11.1b	13.2a	13.6a	12.2ab	11.4bc	10.3c	12.1a	11.9a	11.6a	4.325*	5.880**	0.158 ns	2.450 ns	7.550**	5.376**
Leaf DM(g)	29.9b	40.1a	44.9a	46.4a	39.9ab	36.0ab	30.9b	37.8a	37.9a	39.2a	5.109*	2.589 ns	0.041 ns	0.294 ns	2.469 ns	2.062 ns
Root DM (g)	2.01a	1.79a	2.14a	2.52a	2.07b	1.88bc	1.47c	2.31a	1.82b	1.82b	1.187 ns	8.887**	3.337*	4.014**	8.847**	2.320 ns
IUE_f_ (g liter^−1^)	17.0a	18.8a	15.8a	20.0a	17.4a	15.1a	16.3a	21.5a	17.2b	12.9c	0.986 ns	1.450 ns	14.198**	1.623 ns	2.677 ns	6.178**
IUE_dm_ (g liter^−1^)	6.15b	7.64ab	8.94a	9.75a	7.81ab	6.53b	6.20b	9.23a	7.29b	6.20b	2.827 ns	2.971*	3.524*	4.388**	5.694**	3.996**

Yield is kg per plant for all marketable fruits harvested at 75 day after transplanting (DAT); LAI = Leaf area index measured by canopy solar radiation transmittance method; *LAI*_*xx*_ LAI measured at xx DAT; *DM* g per plant dry matter at 75 DAT; *IUE*_*f*_ or *IUE*_*dm*_ irrigation use efficiency as (marketable fresh fruit yield per plant) or as (g total DM per plant) divided by (liter total irrigation water applied)

**Fig. 1:**
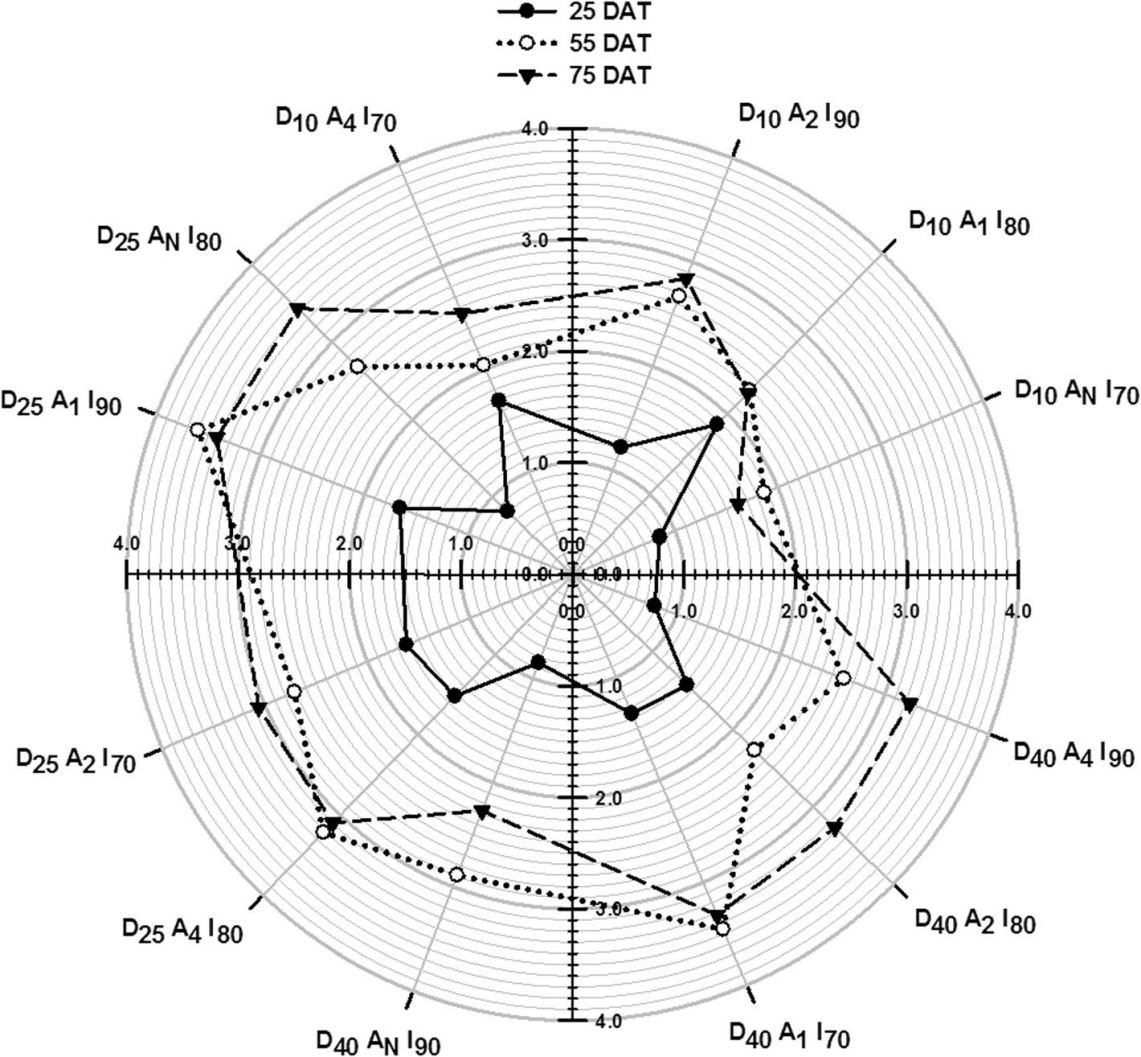
LAI of muskmelon plants measured by canopy solar radiation transmittance at 25, 55, and 75 days after transplanting for each combination of drip-tubing placement depth, soil aeration frequency, and irrigation level treatment factors. Combination treatment means are annotated along polar axis. Ducan _0.05_ for mean comparison at 25, 55, and 75 DAT were 0.50, 1.16, and 1.12

The 1-way ANOVA showed that LAI_25_ values were all < 2.00 for all treatment combinations (Fig. 1). The highest LAI_25_ value of 1.87 for the D_10_A_1_I_80_ treatment combination was significantly higher (P<0.01) than all the non-aerated combinations with values < 1.00 (namely D_10_A_N_I_70_ = 0.81, D_25_A_N_I_80_ = 0.85, D_40_A_N_I_90_ = 0.85) regardless of depth and irrigation level. Treatment combinations with daily soil aeration tended to be higher or similar to those with the 2-day and 4-day soil aeration frequency. Combination with D_40_ and A_4_ gave LAI_25_ < 1. Combinations with higher A_2_ and A_1_ aeration frequency and shallower D_25_ and D_10_ placement depth gave LAI_25_ > 1 indicating that lowering placement depth could offset the negative impact of lowering soil aeration frequency (Fig. 1).

The highest LAI_55_ value of 3.60 during the flowering period was obtained for the D_25_A_1_I_90_. LAI_55_ values > 3.00 were obtained only for the D_25_A_4_I_80_ (= 3.22) and D_40_A_1_I_70_ (= 3.32) treatment combinations. These LAI_75_ values for these combinations were practically the same as those for LAI_55_. It would appear that 3.00 to 3.60 may represent the maximum LAI range for the Shantian No. 1 muskmelon cultivar as measured under the experimental conditions.

Except for the D_10_A_N_I_70_ treatment combination with LAI_55_ = 1.87, all the 8 remaining combination LAI_55_ values were between 2.00 and 3.00. For the D_25_A_1_I_90_ combination, the LAI_55_ = 3.60 represented a 116% increase on the LAI_25_ = 1.66 value. Corresponding increases were 112 and 154% for the D_25_A_4_I_80_ and D_40_A_1_I_70_ treatment combinations. Increases for LAI_55_ relative to LAI_25_ tended to be higher for combinations with the lower (A_4_ and A_N_) soil aeration frequency and especially for the higher irrigation levels (I_90_) (Table 1). As indicated the LAI_75_ values were very close to the LAI_55_ for some combinations indicating little leaf growth after the flowering and during the fruit enlargement growth stages. LAI_75_ values were markedly lower than LAI_55_ for the D_10_A_N_I_70_ and D_40_A_N_I_90_ treatment combinations indicating possible earlier senescence.

#### Dry matter partitioning and irrigation use efficiency of muskmelon

**Fig. 2:**
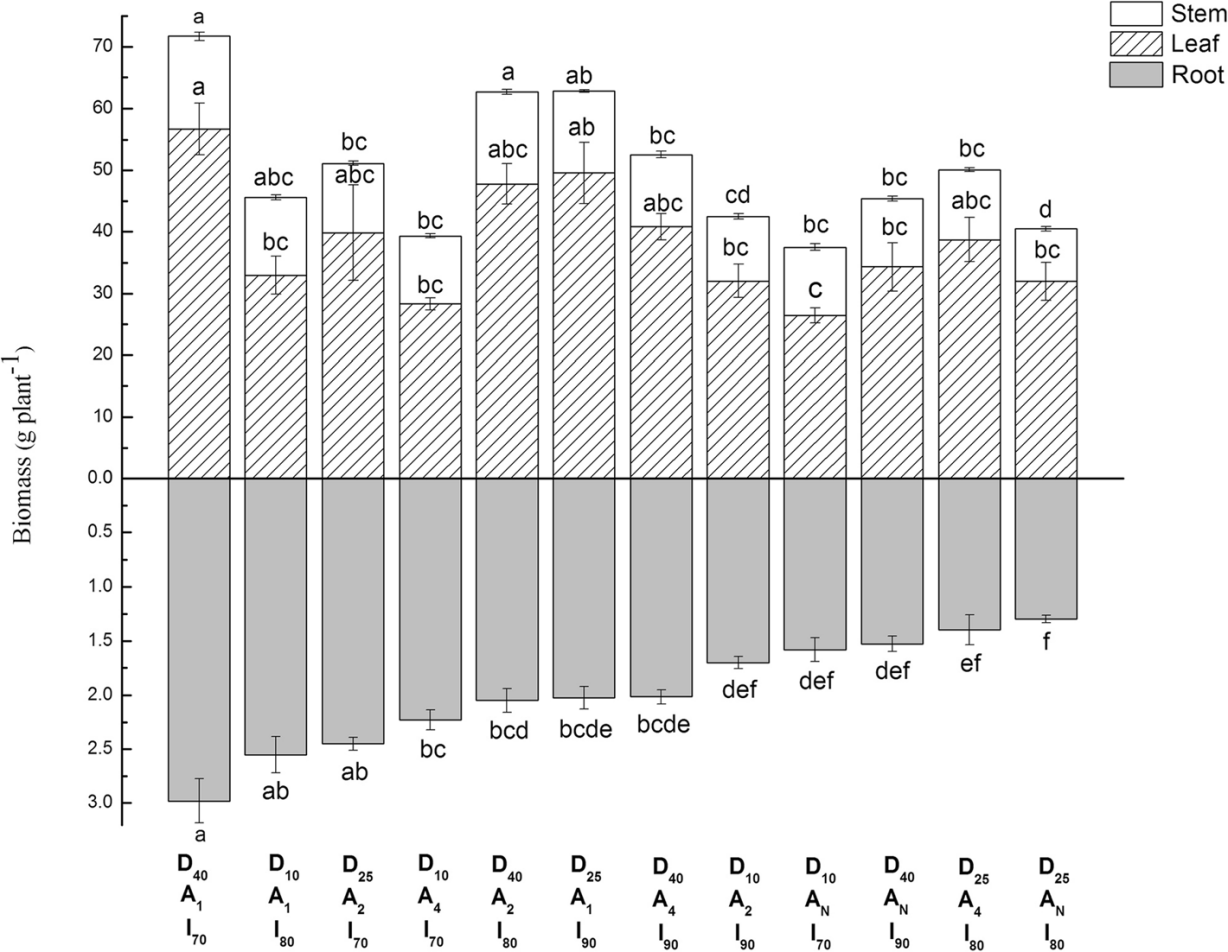
Post-harvest stem, leaf, and root dry matter of muskmelon plants measured at 75 days after transplanting for each combination of drip-tubing placement depth, soil aeration frequency, and irrigation level treatment factors. Bars on the stacked sections for each component represent the standard error of the mean. Means not followed by the same letter are significantly different at the 5% level

Total dry matter of muskmelon progressively increased with increasing tubing placement depth and progressively decreased with decreasing soil aeration frequency (Table 1). Decreased soil aeration frequency tended to progressively reduce all 3 components of the total DM. The effects were more marked for the leaf and root contributions. The marginal mean for leaf and root DM with daily soil aeration was 50 and 71% higher than the values for no aeration. The corresponding value for stem DM was 32%. The marginal mean for root DM was significantly higher for the I_70_ irrigation level compared to the I_80_ and I_90_ levels. In addition, interaction analysis found that D x A and D x I significantly affected the root DM, D x I and A x I significantly affected the Total DM and Stem DM. Overall mean total DM was 52.2 g per plant partitioned as 11.9 g in stem+ 38.3 g in leaf + 2.0 g in root representing 22.7, 73.4, and 3.9% respectively. These DM partitioning percentages were similar for each of the tubing placement depths, soil aeration frequencies, and irrigation levels. Values ranged between 21 and 26%, 68–75%, and 3–4% respectively for the stem, leaf, and root contribution to the total DM for all levels of the treatment factors (Table 1). The D_40_A_1_I_70_ treatment combination resulted in the maximum plant root, stem, leaf and total DM values (Fig. 2). As shown, the plant root, stem, leaf and total DM values for treatment combinations with daily soil aeration tended to be higher than those with the no aeration.

**Fig. 3:**
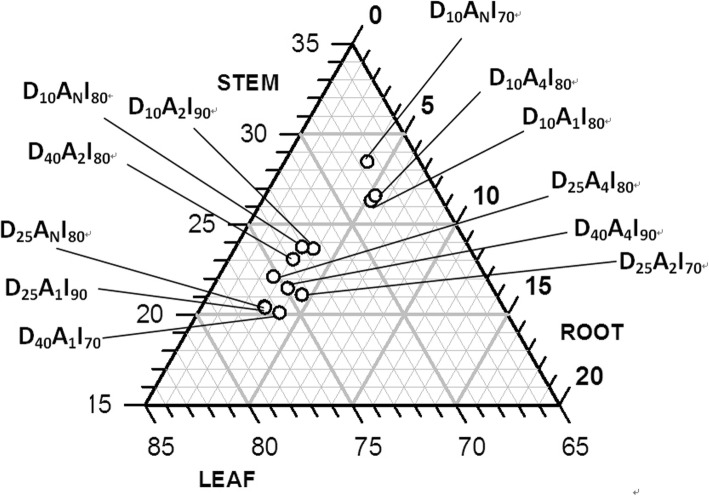
Ternary plot of root, stem, and leaf dry weight of muskmelon expressed as % of total post-harvest plant dry weight measured at 75 days after transplanting foreach combination of drip tubing placement depth, soil aeration frequency, and irrigation level treatment factors

The ternary plot (Fig. 3) show the range of the ratios for root, stem, and leaf DM of muskmelon to total post-harvest DM at 75 days after transplanting for each treatment combination. The root DM percentages fell between 2.8 to 5.4%. The leaf DM range was 67.5 to 75.7% and 20.6 to 28.5% for the stem DM. These ranges were quite narrow indicating that the treatment combinations did not markedly affect DM partitioning in the muskmelon plants. Nevertheless, it would be interesting to examine the clusters of treatment combinations at the high and low ends of these ranges as shown in Fig. 3. The cluster with high stem DM percentages was D_10_A_N_I_70_ > D_10_A_4_I_80_ > D_10_A_1_I_80_ treatment combinations (Fig. 3). The treatment cluster of low leaf DM percentages was D_10_A_N_I_70_ < D_10_A_4_I_80_ < D_10_A_1_I_80_ compared with the cluster of high leaf DM percentages D_25_A_N_I_80_ > D_25_A_1_I_90_ > D_40_A_1_I_70_ treatment combinations. High root DM percentages was obtained for the D_10_A_4_I_80_ > D_10_A_1_I_80_ cluster of treatment combinations. These clusters tended to confirm the existence of 2-factor interactions (Table 1).

The overall irrigation use efficiency for marketable fruit yield of muskmelon (IUE_f_) across each treatment factor in Table 1 was 17.2 g per plant per liter of total irrigation water applied (subsurface drip + initial 70 mm surface irrigation immediately at prior to transplanting). Neither tubing placement depth nor soil aeration resulted in significant differences in the IUE_f_ marginal means. The IUE_f_ marginal mean was 21.5 g fresh fruit per plant per liter for the I_70_ irrigation level. Corresponding values decreased linearly by 20 and 40% for I_80_ (=17.2) and I_90_ (=12.9). These decreases were significantly (*p* < 0.05) different. The 3-way ANOVA indicated a highly significant 2-factor A x I interaction effect (Table 1).

The overall irrigation use efficiency (IUE_dm_) for total dry matter (stem + leaf + root) of muskmelon across each treatment factor in Table 1 was 7.58 g per plant per liter of total irrigation water applied. All treatment factors significantly affected IUE_dm_ and all 2-factor interactions were highly significant (Table 1). The IUE_dm_ value for D_40_ (=8.94) was significantly higher than for D_10_ (=6.15) by 45%. Similarly, IUE_dm_ progressively increased with increasing aeration frequency from 6.20 for A_N_ to 6.53, for A_4_, to 7.81 for A_2_, and 9.75 for A_1_. The 57% increase between the values for A_N_ and A_1_, and the 49% increase between A_4_ and A_1_, were significant (*P* < 0.05). IUE_dm_ values decreased with increasing irrigation level. The marginal mean of 9.23 for I_70_ differed significantly from to the value of 7.29 for I_80_ (20% lower), and 6.20 for I_90_ (33% decrease).

#### Marketable fresh fruit yield of muskmelon

The results in Table 1 show significant treatment main effects for the drip tubing placement depth and soil aeration frequency experimental factors. Increasing depth of tubing tended to decrease the marketable fresh fruit yield per plant in a non-linear manner. Values increased non-significantly from 1.48 to 1.59 kg per plant between the 10 cm to 25 cm tubing placement depth. However, the mean of 1.38 kg per plant for the 40 cm placement depth was significantly lower than for the 25 cm depth (Table 1). Only the D x I two-factor interaction on fresh fruit yield was significant (Table 1) indicating that the tubing placement depth effect would be different for the various irrigation levels. This was due to tubing placement depth determine the position of the soil wetted. Uneven redistribution of soil water in different longitudinal position result in different water use efficiency and fruit yield. It seems that the optimal installation depth of drip pipes is around 25 cm. However, the fractional factorial design does not permit examination of the simple effects of placement depth at each irrigation level.

Yield progressively decreased with decreasing soil aeration frequency from 1.64 kg/plant with daily aeration to 1.51, 1.43 1.35 with 2-day, 4- day, and no aeration, respectively (Table 1). A comparison of means showed that although the daily value was not significantly better the 2-day frequency, it was significantly better than the 4-day and no aeration treatment (Table 1). Irrigation means were not significantly different. Values were 1.53 kg/plant for irrigating to 70% of field capacity and 1.51 and 1.40 kg/plant for the 80 and 90% of field capacity levels (Table 1).

**Fig. 4:**
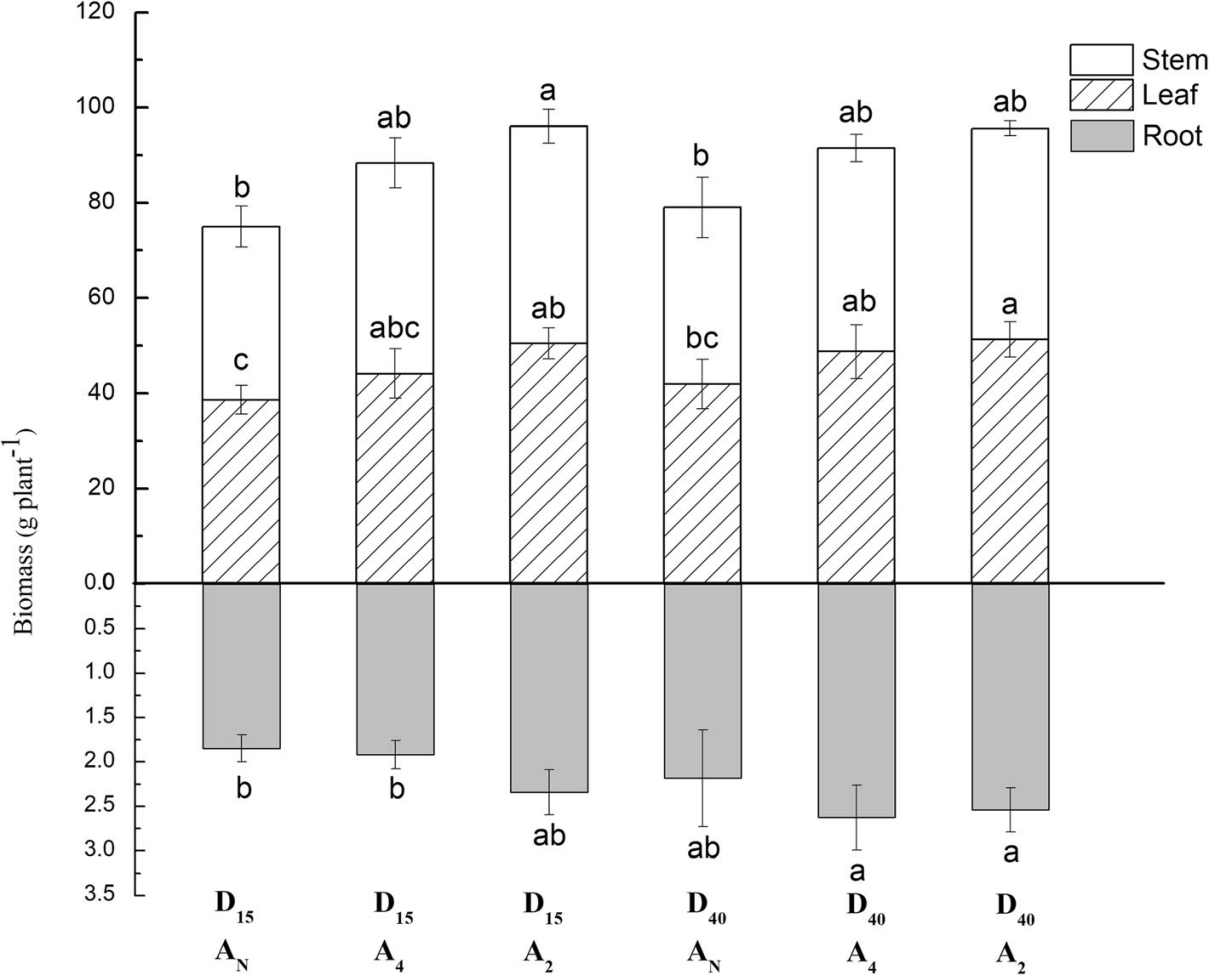
Marketable fruit yield of muskmelon measured for each combination of tubing placement depth (D_cm_), aeration frequency (A _interval in day_), and irrigation level (I _to% field capacity_) treatment factors. Ducan_0.05_ for combination treatment mean comparison = 0.40 kg

The one-way ANOVA for marketable yield supported the foregoing results. When arranged in descending order, the marketable yield in kg per plant tended to fall into several groups for the 12 treatment combinations (Fig. 4). A high-value group comprising in order D_25_A_2_I_70_ (1.67 kg per plant) > D_10_A_1_I_80_ (1.65) = D_40_A_1_I_70_ (1.65) > D_25_A_1_I_90_ (1.63); an intermediate group D_25_A_4_I_80_ (1.54) > D_25_A_N_I_80_ (1.50) > D_10_A_2_I_90_ (1.48); a low yield group D_10_A_4_I_70_ (1.40) > D_10_A_N_I_70_ (1.38) > D_40_A_2_I_80_ (1.37) > D_40_A_4_I_90_ (1.34). The lowest yield of 1.16 kg per plant was obtained for D_40_A_N_I_90_. The high values group included all the high frequency (A_1_) soil aeration. The lower frequency aeration (A_2_, A_4_, A_N_) and the higher irrigation level means (I_80_ and I_90_) were associated with the low value group. These results tend to confirm a positive effect of soil aeration.

Given a measured 4 to 5 fruits per plant, the foregoing results imply increasing fruit size for the grouping of the treatment combinations. Average fresh fruit weight ranged from 232 to 290 g for the lowest yield of 1.16 ± 0.08 kg for D_40_A_N_I_90_. The range for the low value group average of 1.37 ± 0.20 kg was 275 to 343 g, for the intermediate group with 1.41 ± 0.29 kg was 300 to 376 g, and for the high value group with 1.65 ± 0.43 kg was 330 to 412 g. The overall mean across all treatment factors in Table 1 and treatment combinations was 1.48 kg per plant giving an overall average fresh fruit weight ranging between 300 to 360 g for the Shantian No. 1 cultivar.

### Tomato results

#### Dry matter partitioning of tomato

**Fig. 5:**
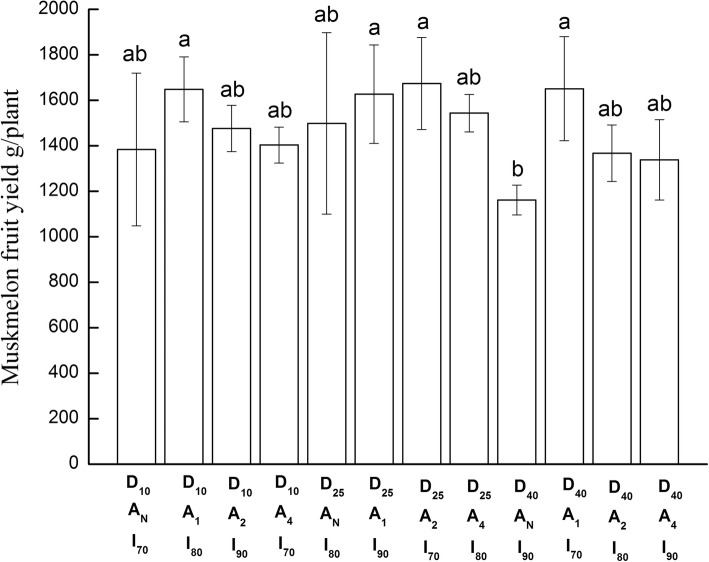
Post-harvest stem, leaf, and root dry matter of tomato measured for each combination of tubing placement depth (D_cm_) and aeration frequency (A _interval in day_) treatment factors. The differences were compared using the Duncan’s test with a significance level of 0.05

Figure 5 shows that Post-harvest stem, leaf, and root dry matter of tomato was greater with aeration than without aeration treatment. For both tube burial depths, Post-harvest stem, leaf, and root DM increased with increasing aeration frequency. Maximal values for stem, leaf, and root DM were obtained in the D_15_A_2_, D_40_A_2_, D_40_A_4_ treatments, respectively. Our investigation found that root DM of tomato with a 40-cm burial of the drip irrigation tube was higher than 15-cm treatment. The highest Total dry matter (stem + leaf + root DM) value of 98.4 g was obtained for the D_15_A_2_.

#### Marketable fresh fruit yield of tomato

**Fig. 6:**
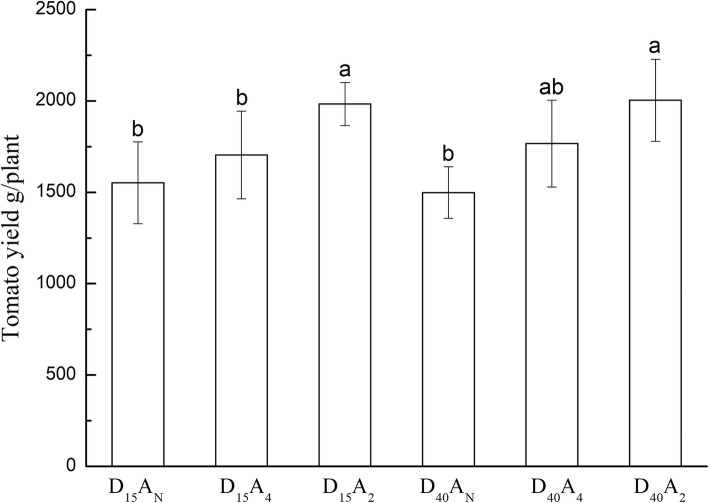
Fruit yield of tomato for each combination of tubing placement depth (D_cm_) and aeration frequency (A _interval in day_) treatment factors. The differences were compared using the Duncan’s test with a significance level of 0.05

Soil aeration improved plant root growth environment, thus increasing yield. Fig. 6 shows that the tomato yield was significantly different between aeration treatments. The results are found for the yield as aeration treatments had significantly higher yield than CK. The D_15_A_2_ treatment combination resulted in the maximum tomato yield (Fig. 6). The D_15_A_2_ was increased the tomato yield by 27.8% compared with the D_10_ A_N._

## Discussion

Sub-surface drip irrigated systems are managed to optimize soil water availability, accessibility, and use efficiency for maximum growth and marketable yield. On the other hand, irrigation always results in displacement and/or redistribution of soil air. This can reduce the level and availability of O_2_ which is continuously needed to act as the electron acceptor in the tricarboxylic acid metabolic cycle, ATP production, and normal root cell activity [22, 23]. Oxygen-deprivation destroys mitochondria and proteins in root cells leading to cytoplasm acidosis and plant growth inhibition [24]. In this context, it would be expected that any negative growth impact of increasing irrigation application can be offset by soil aeration delivered via the drip-irrigation tubing when placed at a given depth in the soil.

### Effect of soil aeration on muskmelon and tomato growth

Hypoxia induces a shift from normal respiration to anaerobic respiration. Previous studies have shown that artificial aeration significantly improved dry matter accumulation, yield, and water use efficiency of muskmelon cotton, wheat, tomato and pineapple [5, 25, 26]. Our results showed that soil aeration had a positive effect on both muskmelon and tomato plant growth. Both muskmelon and tomato DM increased with increasing aeration frequency indicating that rhizospheric oxygen stress do exist, and soil aeration would more likely ameliorate hypoxic conditions.

Applying 405 L of air through the average of 36 emitters would imply soil ventilation at a rate of about 11 L per emitter. It would be expected that most of this air would move upwards by convection displacing existing gases in the air-filled porosity above the plane of the drip lines. In short, the aeration treatments would in effect flush this air-filled porosity. This flushing effect would impact a larger volume of soil with increasing tubing placement depth. Permanent or semi-permanent sub-surface drip irrigation systems need to be placed at depths that permit mechanical surface cultivation. This would reduce the potential of hypoxia. The air-filled porosity in a given soil volume would increase as water is removed by evapo-transpiration. More frequent flushing would increase O_2_ available for root metabolic activities and promote root growth.

Increasing levels of aeration frequency would supply more O_2_ for plant aerobic respiration and generally this would positively increase growth and yield. Any positive effect of aeration increasing soil ventilation on root growth would translate into improved yield and growth as reflected in the consistently higher values of yield, LAI, and DM with increasing soil aeration frequency. Aeration frequency significantly increased tomato growth performance and fruit yield (Table 1). As expected, increasing aeration frequency progressively increased dry matter and marketable fresh fruit yield of tomato (Fig. 5, Fig. 6). This study indicate that soil aeration can significantly increase the plant growth performance. It is consistent with previous studies of tomato, pepper and potato [27–30]. In addition, the aeration treatments tended to significantly impact most responses measured after 25 DAT. Although soil aeration did not significantly affect the LAI_55_ and LAI_75_, the leaf DM response was significant. The reason for this is most likely due to uncontrollable factors (such as solar angle, cloudiness, etc.) that increase the variability in the measured LAI by the canopy solar radiation transmittance method.

### Effect of irrigation on muskmelon growth

The plots were drip-irrigated at 23 and 60 DAT based on the measured gravimetric water content averaged over the 0–60 cm depth of the soil profile on these dates. Based on the volumetric water content of 0.24 measured at transplanting there would be an additional 100 mm x (0.24–0.13) = 11 mm of plant available water in the 50–60 cm profile depth. This would imply a total of 136 mm when added with the 125 mm made available in the 0–50 cm from the surface irrigation at the time of transplanting. This stored water would support an evapo-transpiration rate of close to 6 mm per day over the 0–23 DAT period. This implies there was very likely no soil water deficits during this period. Any effect of the deficit drip irrigation levels at the different tubing placement depths would therefore be more apparent for responses measured after 25 DAT.

At 23 and 60 DAT the plots were drip irrigated with a volume equivalent to the amount required to replenish the measured soil water storage in the 0–60 cm profile depth to 70, 80, or 90% of field capacity. For the I_70_ treatment, this would imply making plant available water in the soil wetted volume around the emitters equal to 600 x (0.7 × 0.38–0.13) mm = 82 mm for both the 37-day period between 23 and 60 DAT, and for the 15-day period between 60 DAT and harvest at 75 DAT. Corresponding values were 104 mm for I_80_ and 127 mm for I_90_.

The physical and hydraulic properties of the deep, loess-derived clay loam soil used in this study were quite uniform with depth. Therefore, it would be expected that the shape and volume of the wetted soil around each emitter would be similar for all tubing placement depths. The shape and dimensions of the wetted soil around the buried emitters would be important in trying to explaining the growth responses to the subsurface drip irrigation treatments for different tubing placement depths (Table 1 and Figs. 1, 2, 3, 4).

In this study, the emitters were spaced 30 cm along the tubing and the tubing was placed 50 cm apart. The foregoing review indicates that the entire thickness of the soil profile for at most 13 cm above and 13 cm below the plane of the drip line placement depths would be wetted by drip irrigation. This thickness would increase with increasing irrigation level. These literature results relating to a soil with the same texture as that used in this study, along with the non-significant effects of irrigation level on most of the post-25 DAT responses (Table 1), tend to confirm that soil water was not a limiting factor in this experiment. The marketable fruit yield, LAI_55_, and LAI_75_ remained unaffected by irrigation level (Table 1, Figs. 1 and 2). For all the measured dry matter accumulation responses at 75 DAT, only the root DM means for the I_80_ and I_90_ irrigation levels were significantly lower compared to the I_70_ level mean (Table 1 and Fig. 2). The irrigation use efficiency for both fruit yield (IUE_f_) and total DM accumulation (IUE_dm_) decreased significantly with increasing irrigation level (Table 1). This would imply that irrigation water applied above the I_70_ level did not contribute to growth and yield since there was no effect of irrigation level on fruit yield and total DM accumulation.

### Effect of burial depths of subsurface tubing on muskmelon and tomato growth

Increasing the placement depth for a given irrigation/aeration level would correspondingly change the position of the soil moisture or soil O_2_ around the emitter. The shape of the wetted volume is expected to change with depth also in homogenous soils due to the effects of transpiration, evaporation and/or ground water. In addition, in soils with marked changes in physical and hydraulic properties with depth increasing the emitter depth would also induce changes in the shape of the wetted volume. Both muskmelon and tomato are moderate-rooted plant, the roots are mainly distributed within 40 cm of the soil surface. The results showed that muskmelon plant DM increased with increasing tubing placement depth (Fig. 2, Table 1). We speculate that lower dry matter accumulation at a shallow depth (10–25 cm) of aeration/ irrigation lines due to the chimney effect. For the root zone soil texture used in this study, the experimental results, in their entirety, tend to support this reasoning. In addition, Wetting the profile 13 cm above and 13 cm below the plane of the drip line placement would imply increasing potential water losses due to soil evaporation for the shallow placement depth with D_10_ > D_25_ > D_40_.

### Interactions of soil aeration, burial depths and irrigation on plant growth

Tomato is a moderate rooted plant, the roots are mainly distributed within 40 cm of the soil surface. Until 23 DAT only the soil aeration treatments were applied via the 10, 25, and 40 cm tubing placement depths. Neither irrigation levels or tubing placement depth had any effect on the marginal mean for LAI_25._ Any effect of these 2 treatment factors would be apparent in the LAI measured at 25 DAT. Nevertheless, the 2-factor D x A interaction was significant affected LAI_25_ (Table 1). On the other hand, all the soil aeration treatments positively affected LAI_25_ compared to the no aeration treatment. This suggests that low natural soil aeration may be a limiting factor in the clay loam soil used in this study. It confirms that artificial ventilation would positively affect growth, and provides more support for studies showing beneficial effects of this practice as was reported by Bhattarai et al. [17] in his comprehensive review of soil aeration research. According to our studies we found that only the D x I two-factor interaction was significant affected muskmelon fruit yield (Table 1). This suggests that tubing placement depth determine the position of the soil wetted. The difference of rhizosphere soil moisture results in the plants grow differently. Soil aeration would more likely ameliorate hypoxic conditions above, rather than below, the emitter. If tubing is placed at a shallow depth (10 cm), plant growth and fruit yield might not be improved significantly because of the chimney effect (Table 1). Also, the 2-factor D x A interaction on LAI_25_ and Root DM were significant indicating that the aeration effect may not be independent of application depth in an initially wet soil profile. On the other hand, the significant 2 factor D x I and A x I interactions on IUE_dm_ indicated that the irrigation level effects varied differently for the tubing placement and aeration treatment factors.

The foregoing discussion raises the question as to whether the roots were able to fully access all the plant available water. Also, how could soil aeration influence the IUE and dry matter partitioning? Unfortunately, there are no recent field investigations on the root system development of muskmelon plants and how the roots explore the soil profile. Assuming a similar overall root architecture and distribution for the Hami melon, it was quite possible that some of the total applied irrigation at the I_80_ and I_90_ may not have been accessible to the plants. If so, this would explain the lack of irrigation effect on the growth responses and the significant decreases in IUE_f_ and IUE_dm_ with increasing irrigation level (Table 1, Figs. 1, 2, 3).

In general, all management practices to optimize the root environment can only indirectly influence crop growth and yield through their direct effect on the root systems with limits dictated by the species genetics [31]. Muskmelon and tomato plant roots are especially vulnerable to Hypoxia stress O_2_ deficiency in the soil reduces adenosine triphosphate (ATP) production leading to lower uptake and transport of nutrients to the shoot leading to a reduction in leaf growth and photosynthesis [32]. The results of this study on muskmelon add new information to the findings of Bhattarai et al. [18, 33–35] and Sharma et al. [36] that show positive effects of soil aeration on growth and yield of other crops.

## Conclusions

The results of this experiment show that it is feasible to configure installation and operation of commercially-available, permanent or semi-permanent, sub-surface drip irrigation systems to manipulate the root environment and indirectly influence growth and yield of greenhouse-produced muskmelon and tomato. For muskmelon experiment, deeper drip tubing placement permits a wider range of mechanical cultivation practices but can induce O_2_ deficits in the wetted zone around the emitters especially for low-frequency, high-volume irrigation applications. The Total DM of muskmelon at 75 DAT increased with increasing tubing placement depth and aeration frequency, the D_40_ was increased the Total DM by 39.3% compared with the D_10_, and the A_1_ was increased the Total DM by 46.9% compared with the A_N_. The fruit yield of muskmelon and tomato increased with increasing aeration frequency, and 1-d aeration intervals was increased the yield of muskmelon by 21.5% compared with the control, and the D_15_A_2_ was increased the tomato yield by 27.8% compared with the D_10_A_N._ Our results demonstrated that the negative impact of any such deficit on the measured responses can be offset by soil aeration via the drip tubing.

## Methods

### Experimental conditions, setup, and treatments

The muskmelon experiment was conducted in a 108 m long and 8 m wide greenhouse located in Yangling, Shaanxi Province, Northwest China (34°17′N latitude, 108°02′E longitude) from April 24 to July 12, 2014. Tomato experiment was conducted from Oct 182,014 to May 20, 2015 in the same greenhouse. The climate is semi-arid with a long-term average annual precipitation of 550–650 mm, with the average annual sunshine of 2163.8 h and 210 frost-free days. The soil texture of root growth area was clay loam with 25.4% sand, 44.1% silt, and 30.5% clay. pH was 7.82, dry bulk density 1.35 g cm^− 3^, porosity 49.4%, and gravimetric field capacity 28.2% (volumetric field capacity 38%). The physical and chemical characteristics of the irrigation water: pH was 7.90, EC was 2.71 ds m^− 1^, total suspended solids were 15 mg L^− 1^, COD_Mn_ was 1.2 mg L^− 1^.

**Fig. 7:**
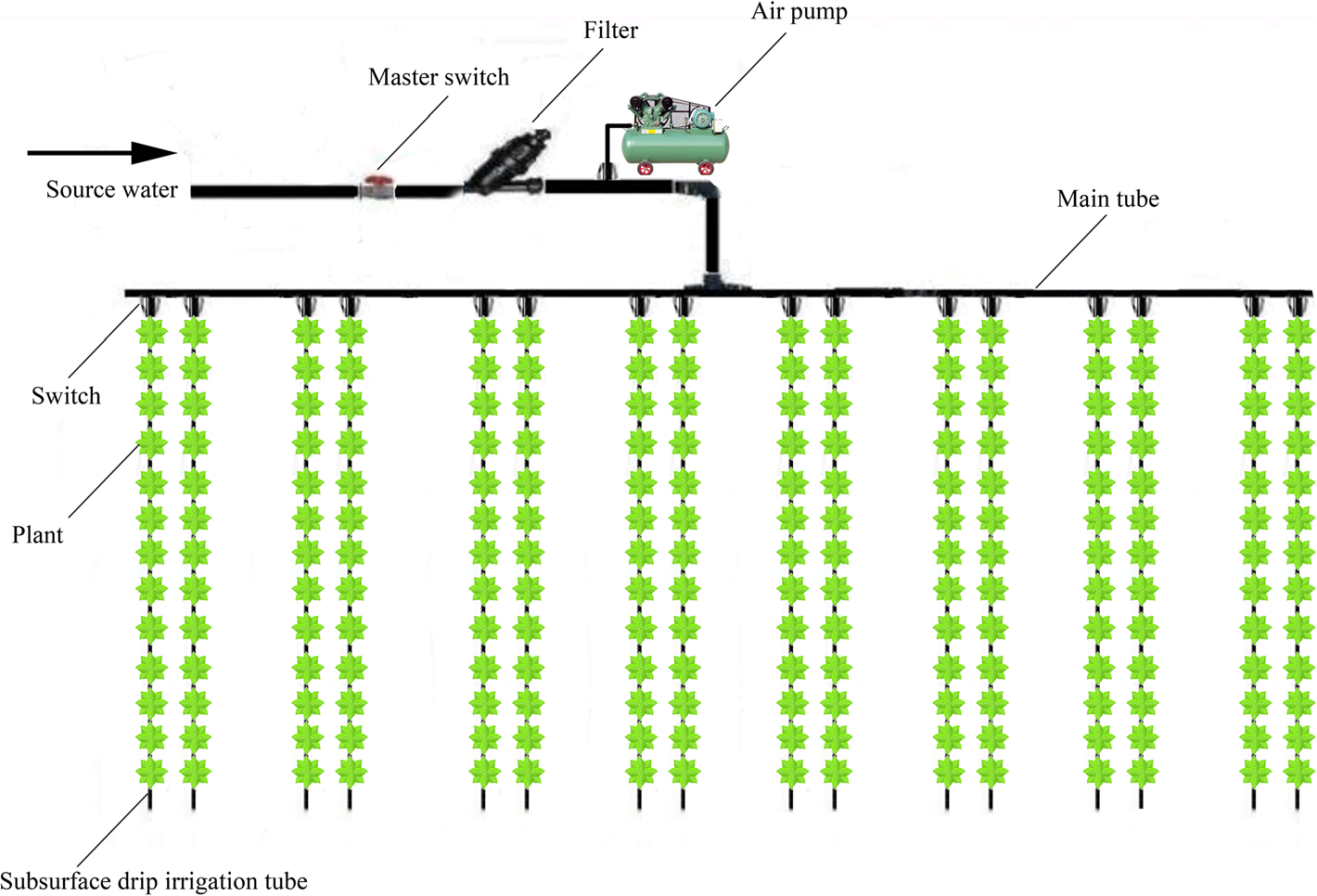
experimental arrangement of an example block

The main drip irrigation tube was connected to an air pump, both water and air were supplied to the soil through the tube (Fig. 7). Drip irrigation tubes (φ16 subsurface drip irrigation pipe, Qinchuan water-saving irrigation equipment engineering Co. Ltd., Yangling, China), 16 mm diameter with emitter spacing of 0.30 m, were buried to the appropriate depth spaced 0.50 m apart along the ridges. The subsurface drip irrigation tubes had a peculiar labyrinth channel structure, so it can promote the uniformity discharge rate of air/water as much as possible. The drip irrigation tubes were connected to a distribution system designed to supply both water and air. Each plot had 35 to 36 emitters.

Muskmelon cultivar Shantian No.1 (Northwest New Horizon Facilities Agriculture Development Co. Ltd., Yangling Shaanxi, China) is one of over 100 cultivars of the Chinese ‘Hami’ reticulated melon group (*Cucumis melo* L.). It is an early maturing, monoecious cultivar with separate male and female flowers on the same plant. Each plant produces 4–5 small (300–350 g), fragrant, sweet, crisp-fleshed, fruits that are very popular with consumers. The tested cultivar of tomato (*Lycopersicon esculentum* Mill.) was Fen-Yu-Yang-Gang (Northwest New Horizon Facilities Agriculture Development Co. Ltd., Yangling Shaanxi, China).

Before transplanting, the soil was rototilled, and 120 t/ha of decomposed organic manure (pig and sheep manure), 400 kg/ha of compound fertilizer (18% N, 15% P_2_O_5_, and 12% K_2_O), and 1500 kg/ha of diammonium phosphate (18% N and 46% P_2_O_e_) were broadcast uniformly in the soil as the basal fertilizer. After 20 days, 26 plugs of muskmelon (or tomato) were transplanted to the experimental plots spaced 0.40 m apart within 2 rows spaced 0.5 m apart on the ridges. To prevent the lateral spread of air and water into adjacent treatments, the plots were separated from each other by a 1.5 m wide empty space. Post-transplanting management practices (i.e. fertilization, agricultural chemicals spraying, fruit pruning etc.) for all plots were consistent with local production practice. Fruiting was not restricted, although some farmers prune the flowers.

Based on the balance between soil air update rate and labor cost, artificial aeration treatments of tomato were none or aeration at 2-day, and 4-day intervals, and interval of aeration for muskmelon were none or aeration at daily, 2-day, and 4-day intervals beginning the first day after transplanting and designated as A_interval in day_ (i.e. A_1_, A_2_, A_4_, and A_N_). For each treatment, 405 L of air was applied to each plot via the drip tubing using a manifold connected to an air compressor. The flow rate for each plot was about 10.2 L min^− 1^. The air which injected via subsurface drip irrigation tubes have a characteristic of high O_2_ concentration and low CO_2_ concentration compared with air which originally stored in soil pore space. The injected air/oxygen consumed by soil microorganisms, soil animals, crop roots. Moreover, a fraction of oxygen diffuses to the atmosphere because of the chimney effect. This volume was calculated as [5, 37]:

$$ {\mathrm{V}}_{\mathrm{A}}=\left[ SL\left(1-{\rho}_{\mathrm{b}}/{\rho}_s\right)\right]/1000\kern0.5em \eta . $$

where *V*_*A*_ was the amount of air injected, *S* the area of a cross-section between rows, *L* the length of plot, *ρ*_b_ the soil bulk density, and *ρ*_*s*_ the soil particle density. *η* an application efficiency coefficient for aeration system.

Irrigation timing and amount demand of tomato experiment is mainly driven by farmers’ perceptions and climatic conditions. For the muskmelon experiment, following recommended production practices the plots were surface-irrigated at the time of transplanting. Soil water content was measured and controlled using a Field TDR 200 soil moisture meter (Spectrum, Aurora, IL, USA). A 60-cm deep probe was installed in the center of each plot. Soil water content was measured at 10-cm intervals down to a depth of 60 cm. The gravimetric water content (θ_g_) averaged over the 0–60 cm at the time of transplanting was measured. Subsurface drip irrigation treatments were based on the measured gravimetric water content averaged over the 0–60 cm depth of the soil profile on these dates. The location of soil sampling was between two rows of muskmelon (or tomato), each soil sampling with three replicates. Muskmelon treatments, designated as I _to% field capacity_ (i.e. I_70_, I_80_, and I_90_), were based on replenishing the water in the soil volume (V_s_) in 60 cm of the soil profile to 70, 80, and 90% of the gravimetric field capacity. The irrigation treatment amount in litre of muskmelon were calculated as: [5, 38]

$$ {V}_I={V}_s\times \uprho b\times \left({\theta}_{gfc}\times {q}_1-{q}_2\right)/\left(\eta \times 1000\right). $$

**Fig. 8:**
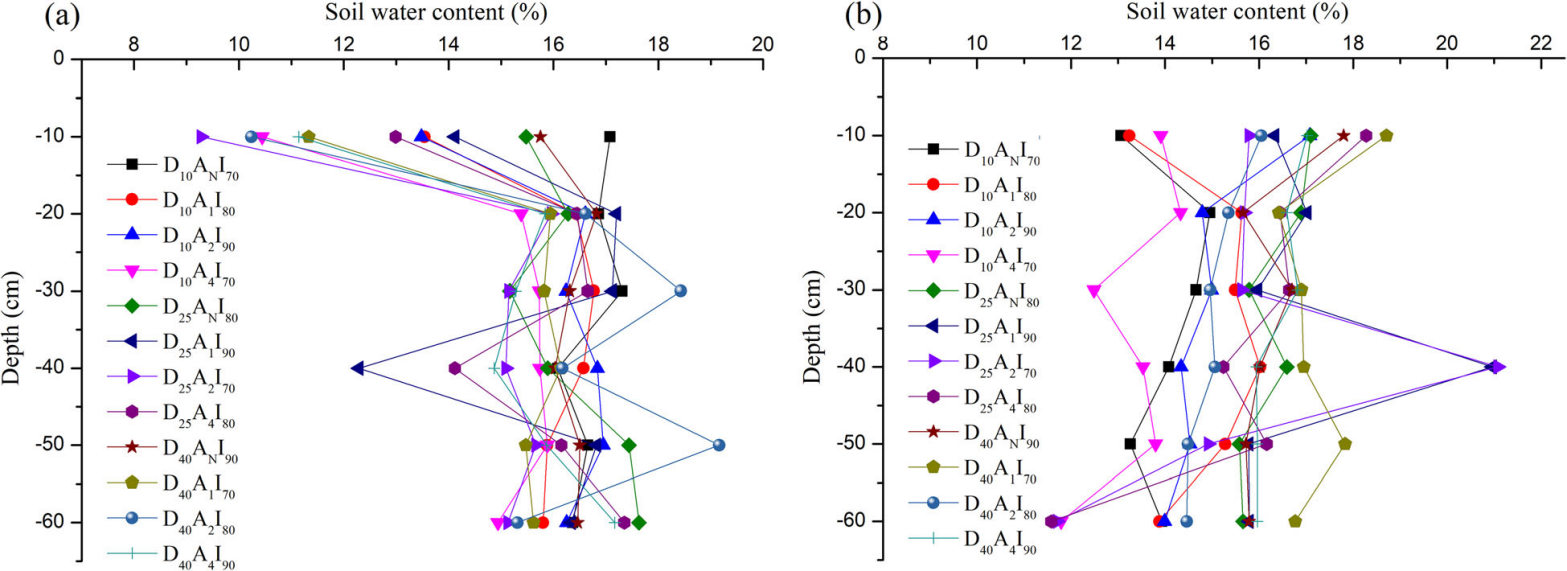
Soil moisture for different treatments and soil depth at (a) 22 days and (b) 59 days after transplanting

Where *V*_*I*_ was the irrigation amount, *ρ*_*b*_ the soil bulk density, *θ*_*gfc*_ field capacity, q_1_ the irrigation level (0.7, 0.8, or 0.9), q_2_ the measured soil moisture content, and *η* an application efficiency coefficient for irrigation system. Irrigation timing demand is mainly driven by farmers’ perceptions and climatic conditions. Details of irrigation timing and amounts during muskmelon growth periods are shown in Table 2. Pre-irrigation soil moisture of muskmelon for different treatments and soil depth at 22 and 59 DAT are shown in Fig. 8.

**Table 2:** Details of irrigation timing and amounts during muskmelon growth period

Combination	Irrigation timing			Total Irrigation amount (mm)
	0DAT (mm)	23DAT (mm)	60DAT (mm)	
D_10_ A_N_ I_70_	70	29.98	53.28	153.26
D_10_ A_1_ I_80_	70	62.21	70.88	203.08
D_10_ A_2_ I_90_	70	84.87	94.29	249.16
D_10_ A_4_ I_70_	70	47.36	59.07	176.43
D_25_ A_N_ I_80_	70	57.33	58.47	185.80
D_25_ A_1_ I_90_	70	88.39	77.01	235.41
D_25_ A_2_ I_70_	70	49.95	37.82	157.77
D_25_ A_4_ I_80_	70	64.03	63.11	197.14
D_40_ A_N_ I_90_	70	82.75	83.00	235.75
D_40_ A_1_ I_70_	70	44.20	27.65	141.86
D_40_ A_2_ I_80_	70	60.88	68.76	199.64
D_40_ A_4_ I_90_	70	93.67	82.16	245.83

*DAT* days after transplanting

### Experimental design

#### Muskmelon experimental design

A full 4^1^ × 3^2^ factorial of the 36 experimental treatment combinations and would require 108 plots for 3 replicates (Table 3). As with all fractional factorial designs, some effects are not estimable and confounding of lower and higher order effects is unavoidable. In this case, the three-factor interaction effect was not estimable. The main effects are independently estimable and it was possible to determine the existence of two-factor interactions. Even with these limitations, the design provided good stability and permitted estimation of the effects of primary interest comparable to the output of the much more expensive complete factorial experiment requiring 108 experimental plots.

**Table 3:** Muskmelon experimental design

Combination	1	2	3	4	5	6	7	8	9	10	11	12
Depth (D)	D_10_	D_10_	D_10_	D_10_	D_25_	D_25_	D_25_	D_25_	D_40_	D_40_	D_40_	D_40_
Aeration (A)	A_N_	A_1_	A_2_	A_4_	A_N_	A_1_	A_2_	A_4_	A_N_	A_1_	A_2_	A_4_
Irrigation (I)	I_70_	I_80_	I_90_	I_70_	I_80_	I_90_	I_70_	I_80_	I_90_	I_70_	I_80_	I_90_

D_10_, subsurface tubing placement depth at 10 cm; D_25_, subsurface tubing placement depth at 25 cm; D_40_, subsurface tubing placement depth at 40 cm; A_N_, aeration treatments were none; A_1_, aeration at daily; A_2_, aeration at 2-day; A_4_, aeration at 4-day; I_70_ irrigation to 70% of the gravimetric field capacity; I_80_ irrigation to 80% of the gravimetric field capacity; I_90_ irrigation to 90% of the gravimetric field capacity

#### Tomato experimental design

The experiment was limited to 6 treatments arranged as a randomised complete block design with drip irrigation tube burial depth and aeration frequency (Table 4).

**Table 4:** Tomato experimental design

Combination	1	2	3	4	5	6
Depth (D)	D_15_	D_15_	D_15_	D_40_	D_40_	D_40_
Aeration (A)	A_N_	A_2_	A_4_	A_N_	A_2_	A_4_

D_15_, subsurface tubing placement depth at 15 cm; D_40_, subsurface tubing placement depth at 40 cm; A_N_, aeration treatments were none; A_2_, aeration at 2-day; A_4_, aeration at 4-day

### Plant measurements

At 75 days after transplanting (DAT), all marketable fruits of muskmelon were harvested from each plot. And 214 days after transplanting (DAT), all marketable fruits of tomato (3 trusses) were harvested from each plot. The fruits were weighed and the fresh fruit weight per plant calculated. At 25, 55, and 75 DAT leaf area index (LAI) was measured by using AccuPARLP-80 canopy analyzer (Decagon Devices, Pullman, Washington 99,163, USA) based on solar radiation transmittance. After harvesting, the remaining above-ground portion of all plants in each experimental plot were collected. The roots were removed and washed through a sieve. Stem and leaves and root were oven dried at 60 °C for 48 h according to Shao et al., 2008; Ahmadi et al., 2014; Ramírez et al., 2014 [39–41]. Root dry weight was then measured on 3 muskmelon (tomato) plants from each plot.

### Data analyses and statistics

The experimental data were organized in Microsoft Excel 2016. The statistical analyses were performed with the SPSS 22 software package (IBM, Armonk, New York). All figures were constructed using the graphing software OriginPro 9.0 (Origin Lab Corporation, One Roundhouse Plaza, Suite 303, Northampton, MA 01060, USA).

### Muskmelon data

Data are expressed as the mean. Three replicates were used for each experimental determination. Data were analyzed using a residual test method before statistical analysis, and the data met the assumption of homogeneity of variances and followed normal distribution. Mean differences between treatments were assessed by analysis of variance (ANOVA). *Post-hoc* pairwise comparisons of the treatment means were performed using Duncan’s multiple range test. Differences were considered significant at the level of 0.05. The main purpose of the ANOVA was to assess the main effects and the 2-way interactions between drip irrigation tube burial depth, irrigation level and artificial aeration frequency on tomato yield, LAI, post-harvest dry matter partitioning, and irrigation use efficiency. The significances were defined at the level of 0.05 and 0.01.

### Tomato data

The experimental design was taken as a 2 × 3 factorial with 3 replicates. Data were analyzed using a residual test method before statistical analysis, and the data met the assumption of homogeneity of variances and followed normal distribution, and all treatment means were compared for significant differences using the Duncan’s new multiple-range test at level of *P* = 0.05.

## Data Availability

The datasets used and/or analyzed during the current study are available from the corresponding author on reasonable request.

## Abbreviations

EC: Electrical conductivity
DAT: Days after transplanting
LAI: Leaf area index
DM: Dry matter
D_cm_ (i.e. D_10_, D_25_, and D_40_): Irrigation tubing placement depths were 10, 25, and 40 cm below the surface of the ridge
IUE_f_ or IUE_dm_: Irrigation use efficiency as (marketable fresh fruit yield per plant) or as (g total DM per plant) divided by (liter total irrigation water applied)
A_interval in day_ (i.e. A_1_, A_2_, A_4_, and A_N_): Interval of aeration for muskmelon/tomato were none or aeration at daily, 2-day, and 4-day intervals beginning the first day after transplanting
I_to% field capacity_ (i.e. I_70_, I_80_, and I_90_): Muskmelon treatments, designated as, were based on replenishing the water in the soil volume (V_s_) in 60 cm of the soil profile (V_s_ = 5.5 m^2^ × 0.6 m) to 70, 80, and 90% of the gravimetric field capacity
LAI _days_ (i.e. LAI_25_, LAI_55_, and LAI_75_): LAI of muskmelon plants measured by canopy solar radiation transmittance at 25, 55, and 75 days after transplanting.
